# Relationship Between Serum Apelin-13 and Apelin Receptor Levels and the Severity of Disease in Patients Diagnosed with Obstructive Sleep Apnea Syndrome

**DOI:** 10.3390/diagnostics15192461

**Published:** 2025-09-26

**Authors:** Demet Aygun, Nilgün Erten, Ulku Dubus Hos, Mustafa Ibas, Naile Fevziye Misirlioglu, Hafize Uzun

**Affiliations:** 1 Department of Neurology, Faculty of Medicine, Istanbul Atlas University, 34408 Istanbul, Turkey; 2 Department of Neurology, Istanbul Physical Medicine and Rehabilitation Training and Research Hospital, University of Health Sciences, 34480 Istanbul, Turkey; nderten@hotmail.com; 3 Department of Neurology, Istanbul Başakşehir Çam and Sakura City Hospital, University of Health Sciences, 34480 Istanbul, Turkey; drulku61@gmail.com; 4 Department of Otorhinolaryngology-Head and Neck Surgery, Faculty of Medicine, Istanbul Atlas University, 34408 Istanbul, Turkey; ibasmustafa@gmail.com; 5 Department of Medical Biochemistry, Faculty of Medicine, Istanbul Atlas University, 34408 Istanbul, Turkeyhuzun59@hotmail.com (H.U.)

**Keywords:** apelin-13, APJ receptor, hypoxia, insulin resistance, obesity, obstructive sleep apnea syndrome (OSAS)

## Abstract

**Background and Objectives:** Apelin-13 and its receptor (APJ) are increasingly recognized as key regulators of metabolic pathways that may contribute to the pathophysiology of obstructive sleep apnea (OSA) syndrome. This study aimed to investigate the relationship between circulating apelin-13 and APJ levels with disease severity in patients diagnosed with OSA, considering the impact of obesity. **Materials and Methods:** A total of 105 subjects were enrolled: 35 obese patients with OSA, 35 non-obese patients with OSA, and 35 healthy controls. Demographic data, polysomnographic parameters, metabolic markers, Apelin-13, and APJ levels were compared across groups. Patients were further classified as mild-moderate, or severe OSA for subgroup analysis. Correlations between Apelin-13, APJ, BMI, minimum oxygen saturation (Min SaO_2_), and apnea–hypopnea index (AHI) were assessed. ROC analysis was used to examine the potential of Apelin-13 and APJ to predict severe OSA. **Results:** Apelin-13 levels were significantly higher in obese patients with OSA compared to non-obese OSA and controls (*p* < 0.001), whereas APJ levels were lowest in obese OSA subjects. Apelin-13 showed significant positive correlations with BMI (r = 0.63, *p* < 0.001) and AHI (r = 0.33, *p* = 0.005), and a negative correlation with Min SaO_2_ (r = −0.35, *p* = 0.004). Conversely, APJ levels were negatively correlated with BMI (r = −0.60, *p* < 0.001) and AHI (r = −0.40, *p* = 0.002) and positively correlated with minimum SaO_2_ (r = 0.40, *p* = 0.002). In severe OSA, insulin and HOMA-IR levels were significantly higher than in mild-moderate OSA (*p* = 0.02 and *p* = 0.003, respectively). However, there was no significant difference in Apelin-13 and APJ levels by OSA severity category. ROC analysis revealed that neither Apelin-13 nor APJ demonstrated sufficient diagnostic performance to predict severe OSA (AUC = 0.50 and 0.63, respectively). **Conclusions:** Apelin and APJ levels are correlated with key metabolic and hypoxic parameters in OSA, indicating that the apelin/APJ system may play a compensatory role in mitigating hypoxia-induced and metabolic complications. However, neither marker alone provides sufficient predictive value for disease severity, emphasizing the need for further studies to clarify the mechanisms and potential clinical applications of this system in OSA management.

## 1. Introduction

Obstructive sleep apnea (OSA) syndrome is a condition characterized by intermittent blockages in the upper airway during sleep. Due to the increasing prevalence of obesity in recent years, OSA is seen in 14% of men and 5% of women [1]. The gold standard for OSA diagnosis is polysomnography (PSG) [2]. The Apnea–Hypopnea Index (AHI) is a PSG parameter used to determine the severity of the disease. Information about OSA is increasing every day. PSG should be performed on every patient suspected of having OSA. The correlation between AHI and various clinical parameters used in diagnosis and disease monitoring has been a subject of research in recent years [2,3,4].

Apelin is an adipocytokine synthesized and secreted from adipose tissue. In addition, it has been found to enhance nitric oxide (NO)-mediated vasorelaxation in the cardiovascular system and to reduce arterial blood pressure. Moreover, apelin provides a potent and long-lasting positive inotropic effect on the heart without inducing myocardial hypertrophy. It has also been shown that apelin synthesis in adipocytes is stimulated by insulin. Apelin-13, which was first isolated from bovine stomach, is a peptide hormone and the endogenous ligand of the apelin receptor APJ [5,6]. Apelin is widely expressed in the central nervous system (CNS) and in various peripheral tissues, including the lungs, heart, kidneys, white adipose tissue, testes, and uterus [7]. The apelin/APJ system plays an important role in the physiological regulation of the cardiovascular system, metabolism, cell proliferation, apoptosis, and the immune system [8,9].

Apelin was initially discovered through a reverse pharmacological approach, first by identifying its receptor (APLNR, also known as APJ) and subsequently the ligand itself [10]. The apelinergic system receptor is composed of 380 amino acids, has a molecular weight of 42,660 Da, and contains seven transmembrane domains. Due to the widespread peripheral distribution of apelin and its receptor (APJ) in many tissues, many studies use a systemic definition and refer to it as the “apelinergic system”. According to its tissue distribution, APJ mRNA has been identified in the lungs, heart, adipose tissue, small intestine, colonic mucosa, ovary, thyroid gland, and hypothalamus [11,12].

Although the apelinergic system has been associated with several cardiometabolic conditions, studies investigating Apelin-13 and its receptor APJ in patients with OSA remain scarce. The apelin/APJ system has been implicated in respiratory diseases and may influence metabolic and cardiovascular alterations in OSA [13,14]. OSA-related systemic factors may further interact with this pathway [15]. Data on how these biomarkers are influenced by obesity and disease severity in OSA are limited [13,14,15]. Additionally, previous studies in OSA have measured total apelin levels, but Apelin-13 specifically was not assessed.

Given inconsistent findings on circulating Apelin-13 and the unclear effect of obesity, this study aimed to evaluate serum levels of Apelin-13 and APJ in patients with OSA and to examine their relationship with obesity, polysomnographic findings, and OSA severity, with the aim of better understanding the potential role of the apelinergic system in the pathophysiology of OSA.

## 2. Materials and Methods

This study was planned as a prospective case–control study at the Atlas University Faculty of Medicine Neurology Clinic. The study was designed in accordance with Good Clinical Practices protocol and the Declaration of Helsinki. Ethics committee approval was obtained from the Istanbul Atlas University invasive scientific research ethics committee (approval number: E-22686390-050.99-68255, Date: 13 June 2025).

### 2.1. Subjects

This cross-sectional observational study included a total of 105 adults aged 18–80 years, stratified into three groups of 35 participants each. Sample size estimation was performed using one-way ANOVA (fixed effects, omnibus, one-way) power analysis, with a moderate effect size (f = 0.30), significance level (α) = 0.05, and statistical power (1–β) = 0.80. The analysis indicated a minimum requirement of 28 participants per group; therefore, 30 participants were recruited into each group to ensure adequacy and account for potential attrition.

- Obese OSA Group: Thirty patients diagnosed with OSA (apnea–hypopnea index [AHI] > 5 events/hour based on PSG) and body mass index (BMI) > 30 kg/m^2^.
- Non-obese OSA Group: Thirty patients diagnosed with OSA (AHI > 5 events/hour based on PSG) and BMI < 30 kg/m^2^.
- Control Group: Thirty healthy individuals with no known chronic or acute medical conditions, AHI < 5 events/hour based on PSG, and BMI < 30 kg/m^2^, matched to the patient groups by age and sex.

The diagnosis and classification of OSA severity were based on overnight PSG conducted by the American Academy of Sleep Medicine (AASM) Scoring Manual Version 3.2024 [16]. The montage included EEG, EOG, EMG, ECG, airflow (nasal pressure transducer and oronasal thermistor), thoracoabdominal effort belts, and pulse oximetry. The study evaluated the following parameters: Total Sleep Time (TST), Sleep Efficiency (SE), percentage of TST with oxygen saturation < 90% (SpO_2_ < 90%), and Arousal Index (AI). TST was defined as the total duration of sleep recorded during PSG. SE was calculated as the ratio of TST to the total time spent in bed. SpO_2_ < 90% was expressed as the proportion of TST with oxygen saturation below 90%. AI was defined as the number of arousals per hour of sleep. AI is the number of arousals per hour of sleep. Respiratory events were scored using AASM 2024 criteria: apnea was defined as a ≥90% reduction in airflow for ≥10 s, and hypopnea as a ≥ 30% reduction for ≥10 s with ≥3% desaturation or arousal. These definitions were used for the diagnosis and classification of OSA severity.

Epworth Sleepiness Scale (ESS) scores were obtained using the validated self-administered questionnaire to assess daytime sleepiness. Total Sleep Time (TST), Sleep Efficiency (SE), % Time SpO_2_ < 90%, and Arousal Index (AI) were recorded during overnight full PSG following standard protocols. TST was calculated as the total duration of sleep during the recording period. SE was calculated as the ratio of TST to total time in bed. % Time SpO_2_ < 90% represents the proportion of total sleep time with oxygen saturation below 90%. AI was determined as the number of arousals per hour of sleep, in accordance with the American Academy of Sleep Medicine (AASM) scoring criteria.

### 2.2. Inclusion Criteria

Inclusion criteria comprised adults aged 18–80 years with PSG-confirmed data. Patients with OSA were required to be newly diagnosed and untreated (i.e., no prior continuous positive airway pressure (CPAP) or titration therapy). Control participants were required to be free from acute or chronic systemic diseases.

### 2.3. Exclusion Criteria

Exclusion criteria included individuals younger than 18 or older than 80 years, those with malignancies, hepatic or renal dysfunction, active or chronic infections, pregnancy, or absence of PSG evaluation. To maintain homogeneity, obese individuals without OSA were not eligible for inclusion in the control group.

### 2.4. Sample Collection and Measurements

Fasting venous blood samples were drawn between 8 and 10 am after the subjects fasted overnight (10–12 h). Blood samples were drawn from the brachial veins in the brachial fossa and placed into plain tubes and anticoagulant-free tubes. The samples were centrifuged for 10 min at 4000 rpm at +4 °C. Biochemical tests were performed immediately. For the measurement of other parameters, serum aliquots were frozen and stored at −80 °C until further analysis.

Serum apelin-13 levels were assayed using a commercially available enzyme-linked immunosorbent assay (ELISA) kit (Human Apelin-13 ELISA Kit, Elabscience, Houston, TX, USA), with a detection range of 125–8000 pg/mL and a sensitivity of 75 pg/mL. According to the manufacturer’s datasheet, the Human Apelin-13 ELISA kit (Catalog No: E-EL-H0458 Elabscience, USA) has been validated to specifically detect apelin-13 with no significant cross-reactivity to other apelin isoforms (e.g., apelin-17, apelin-36) or related peptides. Similarly, the Human Apelin Receptor (APLNR) ELISA kit (Bioassay Technology Laboratory, Shanghai, China) shows no detectable cross-reactivity with other receptors or peptides.

Serum APJ/APLNR levels were assayed using a commercially available enzyme-linked immunosorbent assay (ELISA) kit (Human Apelin Receptor, APLNR ELISA Kit, Catalog No: E1754Hu, Bioassay Technology Laboratory (BT LAB), Shanghai, China), with a detection range of 15–3000 pg/mL and a sensitivity of 7.37 pg/mL. Briefly, samples and standards were added to pre-coated plates, incubated, washed, and then treated with enzyme-linked antibodies. The colorimetric reaction was developed and quantified using a microplate reader at the specified wavelength. All assays showed intra- and inter-assay CVs below 10%, with duplicate measurements at 5.2% for Apelin-13 and 6.1% for APJ, confirming assay reliability.

Biochemical parameters were determined using enzymatic methods (Architect i2000, Abbott Park, IL, USA). Insulin levels were measured by the electrochemiluminescence immunoassay (ECLIA) method on Roche-Hitachi E170 (Roche/Hitachi MODULAR Analytics Combination Systems, Roche Diagnostics, Indianapolis, IN, USA). HbA1c determination was based on HPLC (Variant Turbo II, Bio-Rad Laboratories, Inc., Hercules, CA, USA). Homeostasis model assessment for insulin resistance (HOMA-IR) is calculated by using the following formula:

HOMA-IR = Fasting Insulin (µU/mL) × Fasting Glucose (mg/dL)/405

## 3. Statistical Analysis

Continuous variables were presented as mean ± standard deviation or median (Q1–Q3), while categorical variables were described using frequency and percentage values. The One-Way ANOVA or Kruskal–Wallis H test was used to assess differences in continuous measurements among the three study groups (obese sleep apnea, non-obese sleep apnea, and control). In cases where significant intergroup differences were detected, Post Hoc Tukey tests were performed for pairwise comparisons. For the evaluation of differences between two groups based on OSA severity (mild-moderate vs. severe), Student’s *t*-test or Mann–Whitney U test was utilized. The Chi-square test was employed to evaluate associations between categorical variables (gender, DM, HT) and the study groups. The Pearson correlation test was used to assess the relationship between BMI and Minimum.

The relationships between Apelin-13, APJ levels, and SaO_2_ (%) were evaluated. To assess the predictive utility of Apelin-13 and APJ for severe OSA, Receiver Operating Characteristic (ROC) curve analysis was performed to determine optimal cut-off values, sensitivity, and specificity. The Area Under the Curve (AUC) with corresponding 95% Confidence Intervals (CI) was calculated. A *p*-value < 0.05 was considered statistically significant. All statistical analyses were conducted using IBM SPSS Statistics version 25.

Correlations between continuous variables were primarily assessed using Spearman’s rank correlation coefficient (ρ) to account for potential non-linear relationships and non-normal distributions. As a sensitivity analysis, Pearson’s correlation coefficient (r) was also calculated for the same variables. All statistical tests were two-tailed, and a *p*-value < 0.05 was considered statistically significant.

## 4. Results

### 4.1. Demographic and Polysomnographic Parameters

The demographic and polysomnographic parameters of sleep apnea patients, categorized by obesity status, and control subjects were statistically evaluated for differences. The mean ± SD, median (25th–75th percentile), and n (%) values for Age, Gender, Minimum SaO_2_ (%), BMI (kg/m^2^), and AHI (/h) are presented in Table 1.

The age of obese patients with sleep apnea was 47.9 ± 11.9 years, while it was 49.3 ± 12.7 in non-obese patients with sleep apnea and 47.8 ± 10.3 in the control group; there was no significant difference in mean age among the groups (*p* * = 0.83). In the obese sleep apnea group, 62.9% of patients were male, compared to 80% in the non-obese sleep apnea group and 57.1% in the control group. There was no significant association between the groups and gender (*p* † = 0.11).

The mean Minimum SaO_2_ (%) was 77.7 ± 7.5 in obese sleep apnea patients and 82.5 ± 5.4 in non-obese sleep apnea patients, compared to 96.7 ± 1.4 in the control group. The difference in mean Minimum SaO_2_ (%) among the groups was statistically significant (*p* * ≤ 0.001), with significant differences observed between all groups (*p* § = 0.001; <0.001; <0.001).

**Figure 1:**
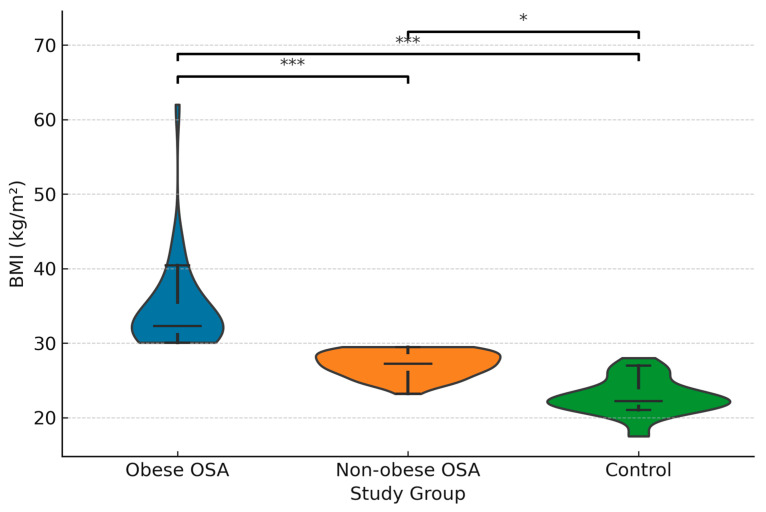
Distribution of body mass index (BMI) across study groups (Obese OSA, Non-obese OSA, Control). Data are presented as violin/box hybrid plots with medians and interquartile ranges indicated. * *p* < 0.05, *** *p* < 0.001.

The mean BMI (kg/m^2^) was 34.6 ± 5.94 in obese sleep apnea patients and 27.16 ± 1.69 in non-obese sleep apnea patients, while it was 22.76 ± 2.37 in the control group. The difference in mean BMI among the groups was significant (*p* * ≤ 0.001), with significant differences found between all groups (*p* § ≤ 0.001; <0.001; <0.001). Figure 1 demonstrates the distribution of BMI across the study groups.

The median AHI (/h) was 54 (28–81) in obese sleep apnea patients, 32 (20–47) in non-obese sleep apnea patients, and 2 (1–2) in the control group. The difference in median AHI (/h) among the groups was significant (*p* ‡ ≤ 0.001), with significant differences observed between all groups (*p* § ≤ 0.001; <0.001; <0.001).

The mean Epworth Sleepiness Scale (ESS) score was 16.17 ± 6.22 in obese sleep apnea patients, 12.57 ± 3.94 in non-obese sleep apnea patients, and 0.35 ± 0.10 in the control group. The difference among the groups was statistically significant (*p* * ≤ 0.001), with significant post hoc differences between all three groups (*p* § ≤ 0.001; <0.001; <0.001).

The mean total sleep time (TST) was 305.06 ± 32.7 min in the obese sleep apnea group, 306.71 ± 34.44 min in the non-obese group, and 427.03 ± 13.24 min in the control group. The difference in TST among the groups was significant (p * ≤ 0.001), with post hoc analysis showing significantly shorter sleep duration in both OSA groups compared to controls (*p* § ≤ 0.001; <0.001), but no significant difference between obese and non-obese patients with OSA (*p* § = 0.21).

The mean sleep efficiency (%) was 74.63 ± 3.17 in obese patients, 78.2 ± 3.68 in non-obese patients, and 85.6 ± 1.15 in controls. The difference in sleep efficiency among groups was statistically significant (*p* * ≤ 0.001), with post hoc comparisons showing significantly lower efficiency in both OSA groups compared to controls (*p* § ≤ 0.001; <0.001).

The mean percentage of total sleep time with SpO_2_ below 90% was 26.4 ± 8.27 in the obese OSA group, 20.91 ± 8.29 in the non-obese OSA group, and 0.05 ± 0.01 in controls. The group difference was statistically significant (*p* * ≤ 0.001), with post hoc analysis showing significant differences across all groups (*p* § ≤ 0.001; <0.001; <0.001).

The mean arousal index was 28.03 ± 7.14 events/hour in the obese OSA group, 20.75 ± 7.24 events/hour in the non-obese OSA group, and 2.91 ± 1.03 events/hour in controls. The difference among groups was statistically significant (*p* * ≤ 0.001), with post hoc analysis confirming significant differences between all groups (*p* § ≤ 0.001; <0.001; <0.001).

### 4.2. Biochemical Biomarkers

The levels of biochemical biomarkers in patients with OSA, categorized by obesity status, and control subjects were statistically evaluated for differences. The mean ± SD and median (25th–75th percentile) values for HbA1c, insulin, glucose, HOMA-IR, APJ (pg/mL), and Apelin-13 (pg/mL) are presented in Table 2.

The mean HbA1c was 6.43 ± 1.42 in obese sleep apnea patients and 6.03 ± 1.11 in non-obese sleep apnea patients, compared to 4.21 ± 0.14 in the control group. The difference in mean HbA1c among the groups was significant (*p* * ≤ 0.001), with the significant difference observed between the control group and both the obese and non-obese sleep apnea patients (*p* ‡ = 0.001; <0.001).

The mean insulin level was 17.69 ± 4.83 in obese sleep apnea patients and 12.21 ± 3.98 in non-obese sleep apnea patients, while it was 15.6 ± 4.33 in the control group. The difference in mean insulin levels among the groups was significant (*p* * ≤ 0.001), with significant differences found between non-obese sleep apnea patients and both the obese sleep apnea patients and the control group (*p* ‡ = 0.001;0.005).

The mean glucose level was 121.57 ± 29.72 in obese sleep apnea patients and 102.94 ± 19.22 in non-obese sleep apnea patients, compared to 92.48 ± 2.91 in the control group. The difference in mean glucose levels among the groups was significant (*p* * ≤ 0.001), with significant differences observed between obese sleep apnea patients and both the non-obese sleep apnea patients and the control group (*p* ‡ = 0.008; <0.001).

The mean HOMA-IR was 5.33 ± 2.47 in obese sleep apnea patients, 3.25 ± 1.74 in non-obese sleep apnea patients, and 1.41 ± 0.86 in the control group. The difference in mean HOMA-IR among the groups was significant (*p* * ≤ 0.001), with significant differences found between all groups (*p* ‡ ≤ 0.001; <0.001; <0.001).

The mean APJ (pg/mL) level was 243.14 ± 17.36 in obese sleep apnea patients, 307.29 ± 10.17 in non-obese sleep apnea patients, and 350.86 ± 17.17 in the control group. The difference in mean APJ levels among the groups was significant (*p* * ≤ 0.001), with significant differences found between all groups (*p* ‡ ≤ 0.001; <0.001; <0.001).

The median Apelin-13 (pg/mL) level was 3970 (3785–4130) in obese sleep apnea patients, 2265 (2117–2360) in non-obese sleep apnea patients, and 1165 (990–1350) in the control group. The difference in median Apelin-13 levels among the groups was significant (*p* † ≤ 0.001), with significant differences found between all groups (*p* ‡ = 0.001; <0.001; <0.001).

### 4.3. Parameters According to OSA Severity

Demographic and polysomnographic parameters were statistically evaluated based on the severity of OSA. The mean ± SD, median (25th–75th percentile), and n (%) values for Age, Gender, DM, HT, Minimum SaO_2_ (%), BMI (kg/m^2^), and AHI (/h) according to OSA severity are presented in Table 3.

There was no significant difference in mean age between patients with mild-moderate OSA (47.1 ± 9.9 years) and those with severe OSA (49.2 ± 13.2 years) (*p* * = 0.51). There was no significant association between OSA severity and gender (65% male in mild-moderate vs. 74% in severe) (*p* † = 0.45), presence of diabetes mellitus (25% in mild-moderate vs. 28% in severe) (*p* † = 0.80), or presence of hypertension (50% in mild-moderate vs. 34% in severe) (*p* † = 0.21). The mean Minimum SaO_2_ (%) was significantly lower in patients with severe OSA (77.8 ± 6.9) compared to those with mild-moderate OSA (85.5 ± 2.4) (*p* * ≤ 0.001). There was no significant difference in mean BMI between the mild-moderate (29.61 ± 3.54) and severe (31.41 ± 6.37) OSA groups (*p* * = 0.24). The median AHI (/h) was significantly higher in patients with severe OSA (48 (36–78)) compared to those with mild-moderate OSA (17 (15–27)) (*p* ‡ ≤ 0.001).

### 4.4. Biomarkers According to OSA Severity

Biomarker levels were statistically evaluated based on OSA severity. The mean ± SD and median (25th–75th percentile) values for these biomarkers are presented in Table 4.

Mean insulin levels were significantly higher in patients with severe OSA (15.71 ± 4.49) compared to those with mild-moderate OSA (12.95 ± 3.79) (*p* * = 0.02). Similarly, the mean HOMA-IR was significantly higher in the severe OSA (4.71 ± 2.53) compared to the mild-moderate group (3.22 ± 1.45) (*p* * = 0.003). There were no significant differences in the mean values of HbA1c, glucose, or APJ (pg/mL) based on OSA severity (*p* * > 0.05). Furthermore, there was no significant difference in the median values of Apelin-13 (pg/mL) based on OSA severity (*p* † > 0.05).

### 4.5. Correlation Analysis

**Figure 2:**
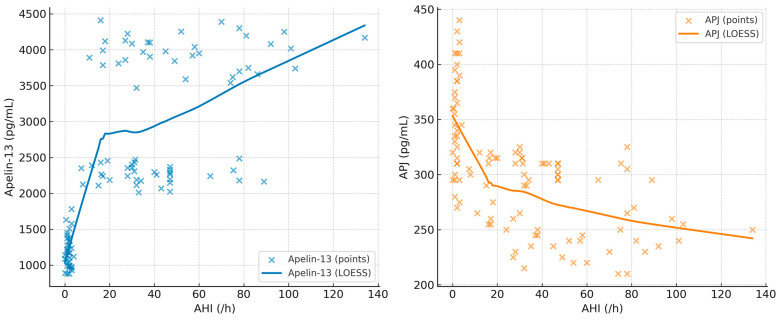
Scatterplots showing the association of AHI (/h) with Apelin-13 (**left**) and APJ (**right**) in patients with OSA. LOESS curves are overlaid to illustrate the non-linear trends.

**Figure 3:**
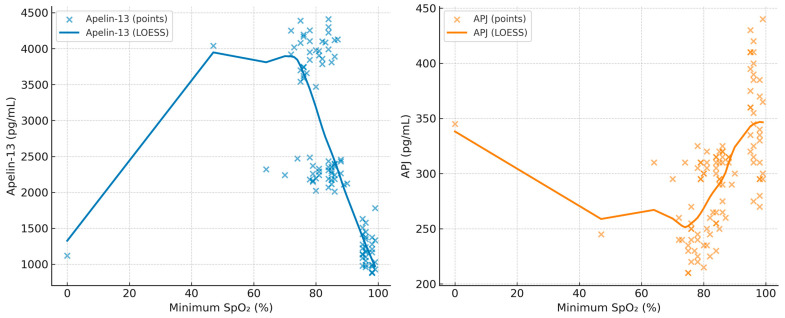
Scatterplots showing the association of minimum SpO_2_ (%) with Apelin-13 (**left**) and APJ (**right**) in patients with OSA. LOESS curves are overlaid to illustrate the non-linear trends.

**Figure 4:**
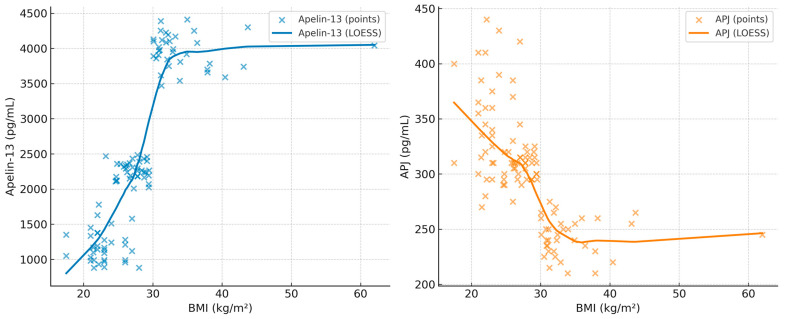
Scatterplots showing the association of BMI (kg/m^2^) with Apelin-13 (**left**) and APJ (**right**) in patients with OSA. LOESS curves are overlaid to illustrate the non-linear trends.

The correlation coefficients between BMI, minimum SpO_2_, AHI, Apelin-13, and APJ are summarized in Table 5. In addition, scatterplots with LOESS smoothing are provided in Figure 1, Figure 2 and Figure 3 to better illustrate the non-linear trends. A significant positive correlation was observed between Apelin-13 and BMI (r = 0.63, *p* < 0.001) and AHI (r = 0.33, *p* = 0.005), whereas a significant negative correlation was found with minimum SpO_2_ (r = −0.35, *p* = 0.004). Conversely, APJ levels showed significant negative correlations with BMI (r = −0.60, *p* < 0.001) and AHI (r = −0.40, *p* = 0.002), and a positive correlation with minimum SpO_2_ (r = 0.40, *p* = 0.002). These associations are visually depicted in Figure 2, Figure 3 and Figure 4, where LOESS curves highlight the non-linear relationships between biomarkers and clinical parameters.

### 4.6. Correlation Between Biomarkers and Hypoxic Burden/Arousal Frequency

Correlation analyses were additionally performed to evaluate the association of serum biomarkers with indices of hypoxic burden and sleep fragmentation in the combined OSA cohort (n = 70). Apelin-13 showed no significant correlation with % time SpO_2_ < 90 (Spearman’s ρ = 0.151, *p* = 0.213), whereas a borderline linear association was observed in the sensitivity analysis using Pearson’s correlation (r = 0.243, *p* = 0.043). In contrast, a significant positive correlation was detected between Apelin-13 and the arousal index (ρ = 0.327, *p* = 0.0058; r = 0.419, *p* = 0.0003). APJ levels exhibited a significant negative correlation with % time SpO_2_ < 90 (ρ = −0.325, *p* = 0.0061; r = −0.342, *p* = 0.0038) and a stronger negative correlation with the arousal index (ρ = −0.497, *p* < 0.001; r = −0.514, *p* < 0.001). These findings are summarized in Table 6.

### 4.7. ROC Analysis for Predicting Severe OSA

**Figure 5:**
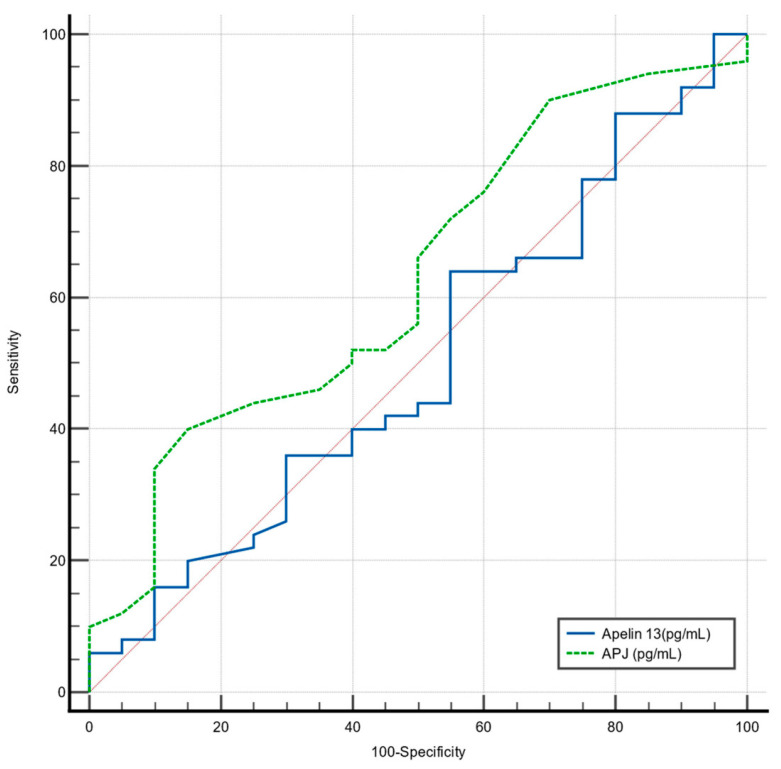
Receiver operating characteristic (ROC) curve for Apelin-13 (pg/mL) and APJ (pg/mL) levels in predicting severe OSA. The red line represents the reference line of no discrimination (AUC = 0.5).

Receiver Operating Characteristic (ROC) analysis was performed to determine cut-off values for Apelin-13 (pg/mL) and APJ (pg/mL) in predicting severe OSA (Figure 5).

For Apelin-13 (pg/mL), the determined cut-off of ≤2455 was not significant for detecting severe OSA, with a sensitivity of 44%, a specificity of 45%, and an Area Under the Curve (AUC) of 0.50 (95% CI: 0.38–0.63; *p* = 0.99). These findings are summarized in (Table 7.)

For APJ (pg/mL), the determined cut-off of ≤250 was also not significant for detecting severe OSA, with a sensitivity of 40%, a specificity of 85%, and an AUC of 0.63 (95% CI: 0.51–0.74; *p* = 0.08).

## 5. Discussion

To our knowledge, this is the first study to simultaneously evaluate both Apelin-13 and its receptor APJ in patients with OSA, with subgroup analyses by obesity status and correlations with polysomnographic and metabolic parameters. We demonstrated that both obesity and OSA severity were associated with significant alterations in metabolic and biochemical parameters. Levels of Apelin-13 were notably higher in obese patients with OSA compared to non-obese patients and healthy controls, while levels of APJ were lower in groups with OSA, particularly in obese individuals. Furthermore, Apelin-13 exhibited a positive correlation with BMI and AHI and a negative correlation with minimum oxygen saturation, indicating its potential role in hypoxia-related processes in OSA. Conversely, APJ levels were inversely correlated with BMI and AHI and positively correlated with oxygen saturation, suggesting that reduced APJ may be linked to disease severity and metabolic dysregulation. However, ROC analysis revealed that neither Apelin-13 nor APJ had sufficient diagnostic value for predicting severe OSA. These findings suggest that Apelin-13 and APJ may reflect metabolic and hypoxic burden in OSA but have limited utility as independent biomarkers for disease severity.

The beneficial roles of Apelin-13 in metabolic disorders indicate that Apelin-13 exerts protective effects in conditions such as obesity and diabetes by improving insulin sensitivity and promoting metabolic homeostasis [17]. In line with this, our study demonstrated significantly elevated Apelin-13 levels in obese patients with OSA, which were positively correlated with BMI, glucose, and HOMA-IR. These findings may reflect a compensatory upregulation of Apelin-13 in response to metabolic dysregulation and insulin resistance in obese individuals with OSA. Therefore, Apelin-13 may act as a metabolic mediator that increases in the presence of both obesity and hypoxia-related stress, although its elevated levels do not necessarily translate into disease severity prediction.

Zirlik et al. [13] measured plasma apelin at four-hour intervals during 24 h diagnostic PSG and again three months after CPAP therapy. Given the strong association of OSA with obesity, hyperinsulinemia, and hypoxia, we measured apelin levels before and after CPAP therapy. In newly diagnosed patients with OSA, plasma apelin was higher than after three months of effective CPAP treatment, showing a trend toward reduction similar to leptin. This change may relate to altered fat distribution, although patient weight remained stable. These findings suggest a potential respiratory role for apelin in chronically hypoxic OSA, warranting further investigation, and are consistent with recent observations by Henley et al. [18] following a glucose challenge.

Apelin is a peptide hormone that plays a significant role in respiratory regulation [19]. At present, CPAP is regarded as an effective treatment for OSA. A previous study has shown that plasma apelin concentrations are elevated in untreated individuals with OSA but tend to normalize following CPAP therapy [18]. This may be attributed to the fact that apelin secretion varies depending on oral glucose load and circadian rhythm. Nizam et al. [14] reported that while serum apelin levels did not differ significantly between OSA and non-OSA individuals, salivary apelin levels were notably higher in patients with OSA, particularly in those with severe disease. The authors suggested that salivary apelin may be modulated locally in the oral cavity in response to inflammation or insulin resistance, rather than solely reflecting circulating levels. Given that both OSA and periodontitis are associated with metabolic dysregulation and insulin resistance, the elevated Apelin-13 levels observed in our study, especially in obese patients with OSA, may similarly reflect a compensatory response to metabolic stress. However, further studies are needed to clarify whether circulating and salivary apelin originate from distinct regulatory mechanisms in OSA [14].

Elevated circulating apelin levels in obese individuals may represent a compensatory response aimed at counteracting insulin resistance, given the metabolic effects of apelin and the absence of marked downregulation of APJ receptor expression in adipose tissue or skeletal muscle. Accordingly, it is plausible that improvements in insulin sensitivity could subsequently result in reduced circulating insulin concentrations [20,21]. In the present study, Apelin-13 levels were significantly elevated in obese patients with OSA and showed a strong positive correlation with insulin resistance indices such as HOMA-IR. Overall, these findings suggest that elevated circulating apelin levels in obese individuals may represent a compensatory response aimed at counteracting insulin resistance, given the metabolic effects of apelin and the absence of marked downregulation of APJ receptor expression in adipose tissue or skeletal muscle. Accordingly, it is plausible that improvements in insulin sensitivity could subsequently result in reduced circulating apelin concentrations. Apelin is a biologically active peptide that binds to its receptor APJ, a G protein–coupled receptor; together, they constitute the apelin/APJ system, which is broadly expressed across various organs and tissues [22].

Apelin receptors are expressed in the lung, and experimental studies have shown that their expression is upregulated under hypoxic conditions [7,23]. In the present study, patients with OSA exhibited significantly lower minimum oxygen saturation, particularly those with severe disease, compared to healthy controls. Notably, APJ concentrations were positively correlated with oxygen saturation, suggesting a potential protective role of the apelin/APJ system in maintaining oxygen homeostasis and mitigating hypoxia-related pathophysiological consequences in OSA. The apelin/APJ signaling pathway has been shown to play a key role in hypoxia-related conditions, with apelin expression upregulated in response to low oxygen levels and contributing to tissue protection under hypoxic stress [24]. The expressions of apelin and APJ are significantly augmented by hypoxia through the hypoxia-inducible factor-1 alpha (HIF-1α) signaling pathway. Therefore, hypoxia induces apelin expression through the activation of HIF-1α. Coincidentally, hypoxia-induced upregulation of HIF-1 provokes the expression of both apelin and APJ in enteric cells and retinal Muller cells [23,25,26]. However, total pulmonary apelin is not altered, but rather increases in correlation with right ventricular pressure in chronic hypoxia [27]. Consistent with these findings, our study observed a positive correlation between APJ concentrations and minimum oxygen saturation in patients with OSA, suggesting that the apelin/APJ system may act as a compensatory mechanism to alleviate hypoxia-related pathophysiological effects. Apelin expression is partially regulated by hypoxia, with upregulation occurring through HIF-1α binding to the apelin gene in various cell types that respond to oxygen deprivation [23]. These results suggest that the apelin/APJ signaling pathway plays a crucial role in maintaining oxygen homeostasis and could be a promising target for alleviating hypoxia-induced complications in OSA.

The present study analyzed biomarkers according to the severity of OSA. The results showed that insulin levels and HOMA-IR were significantly higher in patients with severe OSA compared to those with mild to moderate disease. However, HbA1c, glucose, APJ, and Apelin-13 levels did not differ significantly between the severity groups. Correlation analysis revealed that Apelin-13 was positively associated with BMI and AHI and negatively correlated with minimum oxygen saturation. In contrast, APJ showed an inverse pattern; it was negatively associated with BMI and AHI and positively correlated with oxygen saturation. ROC analysis indicated that neither Apelin-13 nor APJ had sufficient predictive value for severe OSA. Apelin-13, as an adipokine, is upregulated in obesity. In clinical and experimental studies, serum apelin level or its adipose tissue expression is increased in obesity and insulin resistance status [28,29]. Apelin, through the apelin–APJ system, plays a key role in glucose metabolism by enhancing insulin sensitivity, stimulating glucose uptake, and regulating lipid metabolism. Consequently, this system represents a potential therapeutic target for diabetes and its associated complications [30]. Deficiency in the hormone apelin is associated with impaired glucose tolerance, insulin resistance, and increased adiposity. In contrast, the administration of exogenous apelin or apelin analogues has been shown to improve insulin sensitivity, glucose uptake, and metabolic parameters in obese and insulin-resistant mice [31,32,33,34,35,36]. Although the apelin/APJ system is known to influence glucose and lipid metabolism, its precise mechanisms, signaling pathways, and potential adverse effects, particularly in humans, remain incompletely understood, highlighting the need for further research on receptors, ligands, and specific apelin isoforms. These findings suggest that while the apelin/APJ system plays a central role in metabolic regulation, its dysregulation in OSA may contribute to the metabolic and hypoxic complications observed in these patients.

### Strengths and Limitations

This study is the first to simultaneously evaluate circulating Apelin-13 and its receptor APJ in patients with OSA, including subgroup analyses by obesity and correlations with polysomnographic and metabolic parameters. The use of well-validated ELISA kits with low intra- and inter-assay variability (<10% CV) ensured reliable and specific measurement of these biomarkers.

Limitations include the relatively small sample size and cross-sectional design, which limit generalizability and prevent causal inference. Body composition and fat distribution were not assessed, and longitudinal changes following interventions such as CPAP were not examined. An apparent gap in Apelin-13 values (2500–3500 pg/mL) likely reflects inter-batch ELISA variability rather than true biological absence. Additionally, potential confounders such as diet, physical activity, comorbidities, and medications were not fully controlled.

Despite these limitations, our findings demonstrate that the apelin–APJ system is closely linked to key metabolic and hypoxic parameters in OSA, with Apelin-13 correlating positively with BMI and AHI, and APJ levels reflecting oxygen saturation. This suggests a compensatory role in counteracting the metabolic and cardiovascular consequences of chronic intermittent hypoxia. Although neither marker alone reliably predicts disease severity, these results highlight the potential of targeting the apelin–APJ system as a treatment target. Future studies with larger cohorts, longitudinal follow-up, and detailed evaluation of apelin isoforms are warranted to clarify their mechanistic and clinical relevance in OSA management.

## Figures and Tables

**Table 1:** Demographic and polysomnographic parameters of sleep apnea patients by obesity status and controls.

	Obese (1) *n* = 35	Non-Obese (2) *n* = 35	Control (3) *n* = 35	*p* Value	*p* Value § 1 vs. 2 1 vs. 3 2 vs. 3
Age (years), mean ± SD	47.9 ± 11.9	49.3 ± 12.7	47.8 ± 10.3	0.83 *	-
Gender (male), *n* (%)	22 (62.9)	28 (80)	20 (57.1)	0.11 †	-
Min SaO_2_ (%), mean ± SD	77.7 ± 7.5	82.5 ± 5.4	96.7 ± 1.4	<0.001 *	0.001; <0.001; <0.001
BMI (kg/m^2^), mean ± SD	34.6 ± 5.94	27.16 ± 1.69	22.76 ± 2.37	<0.001 *	<0.001; <0.001; <0.001
AHI (/h), Median (Q1–Q3)	54 (28–81)	32 (20–47)	2 (1–2)	<0.001 ‡	0.001; <0.001; <0.001
Epworth Sleepiness Scale, mean ± SD	16.17 ± 6.22	12.57 ± 3.94	0.35 ± 0.10	<0.001 *	0.02; <0.001; <0.001
Total Sleep Time (min), mean ± SD	305.06 ± 32.7	306.71 ± 34.44	427.03 ± 13.24	<0.001 *	0.98; <0.001; <0.001
Sleep Efficiency (%)	74.63 ± 3.17	78.2 ± 3.68	85.6 ± 1.15	<0.001 *	0.001; <0.001; <0.001
% Time SpO_2_ < 90%, mean ± SD	26.4 ± 8.27	20.91 ± 8.29	0.05 ± 0.01	<0.001 *	0.009; <0.001; <0.001
Arousal Index (/h), mean ± SD	28.03 ± 7.14	20.75 ± 7.24	2.91 ± 1.03	<0.001 *	<0.001; <0.001; <0.001

* SD: Standard Deviation; Q1–Q3: 25th–75th percentile; One-Way ANOVA; †: Chi-square test; ‡: Kruskal–Wallis H test; §: Post Hoc Tukey test; *p* < 0.05 is significant.

**Table 2:** Biochemical biomarkers according to obesity status in patients with OSA and in controls.

Sleep Apnea	Obese (1) *n* = 35	Non-Obese (2) *n* = 35	Control (3) *n* = 35	*p* Value	*p* Value ‡ 1 vs. 2 1 vs. 3 2 vs. 3
HbA1c, mean ± SD	6.43 ± 1.42	6.03 ± 1.11	4.21 ± 0.14	<0.001 *	0.26; <0.001; <0.001
Insulin, mean ± SD	17.69 ± 4.83	12.21 ± 3.98	15.6 ± 4.33	<0.001 *	0.001; 0.12; 0.005
Glucose, mean ± SD	121.57 ± 29.72	102.94 ± 19.22	92.48 ± 2.91	<0.001 *	0.008; <0.001; 0.21
HOMA-IR, mean ± SD	5.33 ± 2.47	3.25 ± 1.74	1.41 ± 0.86	<0.001 *	<0.001; <0.001; <0.001
APJ (pg/mL), mean ± SD	243.14 ± 17.36	307.29 ± 10.17	350.86 ± 17.17	<0.001 *	<0.001; <0.001; <0.001
Apelin-13 (pg/mL), Median (Q1–Q3)	3970 (3785–4130)	2265 (2117–2360)	1165 (990–1350)	<0.001 †	0.001; <0.001; <0.001

* SD: Standard Deviation; Q1–Q3: 25th–75th percentile; One-Way ANOVA; †: Kruskal–Wallis H test; ‡: Post Hoc Tukey test; *p* < 0.05 is significant.

**Table 3:** Demographic and polysomnographic parameters by OSA Severity.

	Mild-Moderate *n* = 20	Severe *n* = 50	*p* Value
Age (years), mean ± SD	47.1 ± 9.9	49.2 ± 13.2	0.51 *
Gender (male), *n* (%)	13 (65)	37 (74)	0.45 †
Diabetes Mellitus, *n* (%)	5 (25)	14 (28)	0.80 †
Hypertension, *n* (%)	10 (50)	17 (34)	0.21 †
Min SaO_2_ (%), mean ± SD	85.5 ± 2.4	77.8 ± 6.9	<0.001 *
BMI (kg/m^2^), mean ± SD	29.61 ± 3.54	31.41 ± 6.37	0.24 *
AHI (/h), Median (Q1–Q3)	17 (15–27)	48 (36–78)	<0.001 ‡

* SD: Standard Deviation; Q1–Q3: 25th–75th percentile; Student’s *t*-test; † Chi-square test; ‡ Mann–Whitney U test; *p* < 0.05 is significant.

**Table 4:** Biomarkers by OSA Severity.

	Mild-Moderate *n* = 20	Severe *n* = 50	*p* Value
HbA1c, mean ± SD	6.52 ± 1.66	6.11 ± 1.09	0.23 *
Insulin, mean ± SD	12.95 ± 3.79	15.71 ± 4.49	0.02 *
Glucose, mean ± SD	111.55 ± 21.71	112.54 ± 28.29	0.90 *
HOMA-IR, mean ± SD	3.22 ± 1.45	4.71 ± 2.53	0.003 *
APJ (pg/mL), mean ± SD	284.74 ± 12.18	271.40 ± 16.01	0.15 *
Apelin-13 (pg/mL), Median (Q1–Q3)	2442 (2246–3965)	3505 (2263–3972)	0.95 †

* SD: Standard Deviation; Q1–Q3: 25th–75th percentile; Student’s *t*-test; †: Mann–Whitney U test; *p* < 0.05 is significant.

**Table 5:** Correlation Assessment Between BMI, Min SaO_2_ (%), Apelin-13, and APJ in Patients.

	Apelin-13 (pg/mL)		APJ (pg/mL)	
	r	*p*	r	*p*
BMI	0.63	<0.001	−0.60	<0.001
Min SaO_2_ (%)	−0.35	0.004	0.40	0.002
(AHI/h)	0.33	0.005	−0.40	0.002

r: correlation coefficient; Pearson correlation test, *p* < 0.05 is significant.

**Table 6:** Correlation of Apelin-13 and APJ with hypoxic burden and arousal frequency in patients with OSA (*n* = 70).

	Apelin-13 (pg/mL)		APJ (pg/mL)	
	r	*p*	r	*p*
% Time SpO_2_ < 90%	0.151	0.213	−0.325	0.006
Arousal Index (/h)	0.327	0.006	−0.497	<0.001

**Table 7:** Cut-Off Determination for Apelin-13 (pg/mL) and APJ (pg/mL) in Predicting Severe OSA.

	AUC (%95 Cl)	Cut-Off	Sensitivity %	Specificity %	*p* Value
Apelin-13 (pg/mL)	0.50 (0.38–0.63)	≤2455	44	45	0.99
APJ (pg/mL)	0.63 (0.51–0.74)	≤250	40	85	0.08

## Data Availability

The data that support the findings of this study are available on request from the corresponding author. The data are not publicly available due to privacy or ethical restrictions.
